# Mutations at Two Key Sites in PP2A Safeguard *Caenorhabditis elegans* Neurons from Microcystin-LR Toxicity

**DOI:** 10.3390/toxins16030145

**Published:** 2024-03-13

**Authors:** Chunhua Zhan, Jianke Gong

**Affiliations:** College of Life Science and Technology, Key Laboratory of Molecular Biophysics of MOE, Huazhong University of Science and Technology, Wuhan 430074, China; d202080713@hust.edu.cn

**Keywords:** Microcystin-LR, neurotoxic, PP2A, *Caenorhabditis elegans*

## Abstract

Microcystin-LR (MC-LR) is a secondary metabolite produced by cyanobacteria, globally renowned for its potent hepatotoxicity. However, an increasing body of research suggests that it also exhibits pronounced neurotoxicity. PP2A is a fundamental intracellular phosphatase that plays a pivotal role in cell development and survival. Although extensive research has focused on the binding of MC-LR to the C subunit of PP2A, few studies have explored the key amino acid sites that can prevent the binding of MC-LR to PP2A-C. Due to the advantages of *Caenorhabditis elegans* (*C. elegans*), such as ease of genetic editing and a short lifespan, we exposed nematodes to MC-LR in a manner that simulated natural exposure conditions based on MC-LR concentrations in natural water bodies (immersion exposure). Our findings demonstrate that MC-LR exerts comprehensive toxicity on nematodes, including reducing lifespan, impairing reproductive capabilities, and diminishing sensory functions. Notably, and for the first time, we observed that MC-LR neurotoxic effects can persist up to the F3 generation, highlighting the significant threat that MC-LR poses to biological populations in natural environments. Furthermore, we identified two amino acid sites (L252 and C278) in PP2A-C through mutations that prevented MC-LR binding without affecting PP2A activity. This discovery was robustly validated through behavioral studies and neuronal calcium imaging using nematodes. In conclusion, we identified two crucial amino acid sites that could prevent MC-LR from binding to PP2A-C, which holds great significance for the future development of MC-LR detoxification drugs.

## 1. Introduction

The reoccurrences of cyanobacterial blooms have prompted substantial global apprehension [1,2]. Among the most serious consequences of cyanobacterial blooms is the release of microcystins (MCs) into natural water bodies, with microcystin-LR (MC-LR) being the most widespread and potent [3,4]. Due to its accumulation in food chains, MC-LR poses a substantial threat to the safety of various organisms, ultimately endangering human health [5,6]. Multiple incidents of MC-LR-induced human poisoning have been documented throughout history, resulting in fatalities [7]. In particular, in developing countries, there is less attention on MC-LR, potentially leading to large-scale human poisoning incidents [8]. Consequently, the World Health Organization (WHO) has established a threshold of 1 μg/L for MC-LR in drinking water. However, in natural water bodies, MC-LR concentrations often reach several tens to hundreds of micrograms per liter [9]. Historically, the liver has been identified as the principal organ susceptible to the toxic effects of MC-LR [10,11]. However, recent research has unveiled the severe neurotoxic effects of MC-LR, the underlying mechanisms of which remain unclear [12,13]. Additionally, although the toxicity of MC-LR is well-established, research on detoxification mechanisms is limited. Therefore, in-depth explorations of the toxic mechanisms of MC-LR lay a solid foundation for the development of MC-LR detoxification strategies.

Direct contact and food-chain transmission are the primary modes of exposure for aquatic organisms to MC-LR. In 2008, a *Microcystis aeruginosa* outbreak in Algeria resulted in a significant mortality of sea turtles [14]. Miller et al. (2010) traced MC-LR along the food chain from rivers to a gastropod inhabiting a bay, demonstrating a progressive accumulation and amplification through the biological chain [15]. As a consequence, over twenty sea otters succumbed to poisoning after predating on these gastropods. MC-LR pose a substantial threat to fish populations, exerting significant impacts through both direct toxic effects and the induction of secondary diseases [16,17]. Moreover, wildlife in natural ecosystems is highly susceptible to MC-LR threats. In Tanzania’s Lake Manyara, a massive die-off of lesser flamingos occurred due to MC-LR poisoning [18]. In 1995, a proliferation of MC-LR aeruginosa in a pond in Nishinomiya, Japan, led to widespread mortality among wild birds, including dabbling ducks [19]. In 2001, a sudden mass-mortality event affected flamingos and other waterfowl in Southwest Spain’s Doñana National Park, attributed to the ingestion of toxic cyanobacteria [20]. By July 2004, at least 6000 individuals from 47 different avian species (including endangered ones such as the marbled teal and white-headed duck) had succumbed to MC-LR contamination in Doñana National Park [21]. Recent research indicates that the enigmatic large-scale mortality of bald eagles can be attributed to an outbreak of vacuolar myelinopathy within the bald eagle population. This outbreak was caused by epiphytic cyanobacteria thriving on aquatic vegetation [22]. These instances underscore the profound risk of MC-LR poisoning faced by wildlife populations in natural ecosystems. In the past, several countries have witnessed human poisoning incidents caused by MC-LR. In 1996, exposure to MC-LR-contaminated water at a blood dialysis center in Brazil led to the death of 86 individuals, with acute liver toxicity being the predominant pathological feature [9]. In 2007, an Argentinean male went surfing in a lake affected by a cyanobacterial bloom and ingested contaminated water, subsequently experiencing symptoms such as nausea, abdominal pain, and fever a few days later [23]. Moreover, residents living around China’s Lake Chao Hu, where cyanobacterial blooms frequently occur, have exhibited varying degrees of liver damage. Blood tests have revealed elevated levels of MC-LR in their bloodstream [24,25]. In addition to entering the human body through drinking water and food, recent research has also indicated that MC-LR can be dispersed over long distances through aerosols [26,27]. This suggests that humans are exposed to significant risks of MC-LR exposure. Hence, it is of paramount importance to delve deeper into the detoxification mechanisms of MC-LR.

The primary molecular target of MC-LR is the C subunit of the PP2A protein, which is a heterotrimer with three subunits and a very important phosphatase protein that is functionally conserved and widely distributed in various species [28,29,30,31]. In most cases, MC-LR enters the body from polluted water and food sources; binds to the PP2A protein C subunit in various organ cells such as the liver, intestine, and kidney; causes cell stress, peroxidation, and apoptosis; then further threatens individual life [32,33]. Structural studies have revealed that MC-LR binds to the C subunit, forming covalent bonds and van der Waals forces with six amino acid residues, which are located in the functional motif of the C subunit [28,34]. However, the identification of the key amino acid residues within PP2A-C that interact with MC-LR and that could be potential targets for future drug development remains a mystery. *Caenorhabditis elegans* (*C. elegans*) is a valuable tool to study the molecular mechanisms of environmental toxicants such as MC-LR due to its genetic and neurobiological advantages [35,36]. The worm genome possesses mammalian PP2A homologous genes; the C subunit is *let-92* [37,38].

In this study, we comprehensively assessed the toxic effects of MC-LR on nematodes and demonstrated its accumulation within their bodies. We made the groundbreaking discovery of MC-LR transgenerational neurotoxicity. Through a combination of bioinformatics and gene-editing techniques, we identified two critical amino acid residues that, without affecting PP2A activity, could prevent the binding of PP2A-C to MC-LR. These findings were subsequently validated through in vivo calcium imaging. This study lays the foundation for the future development of detoxification agents for MC-LR and provides essential guidance for water pollution control and ecosystem protection.

## 2. Results

### 2.1. MC-LR Exposure Alters the Lifespan and Reproduction Ability of Nematodes

**Figure 1:**
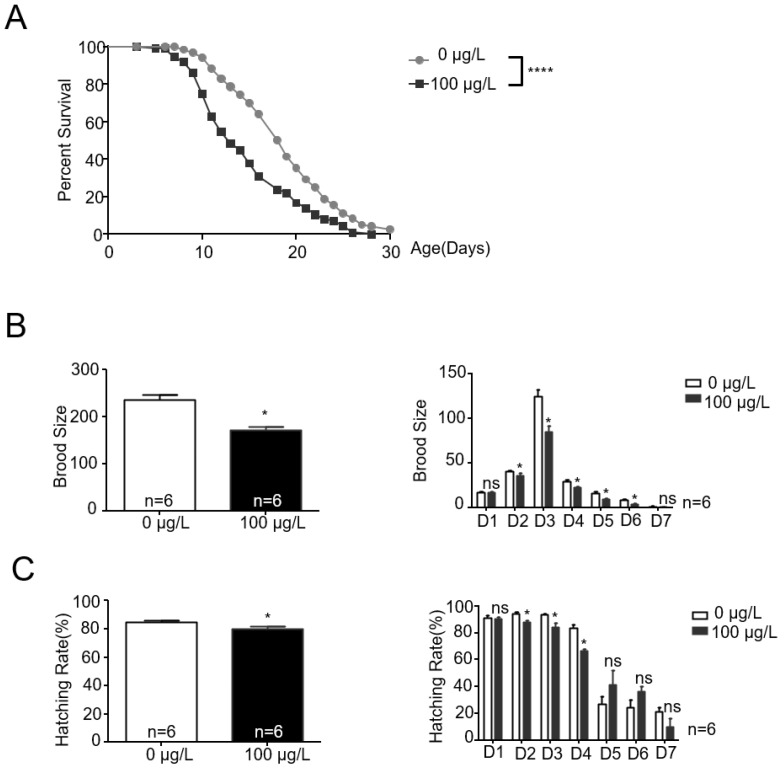
MC-LR exposure alters the lifespan and reproduction ability of nematodes. (**A**) Lifespan curves of exposure and non-exposure animals showing a significant difference. Exposure animals were shorter-lived compared with the control group. The values are presented as the mean ± SEM and were analyzed using a *t*-test (n = 3 per group). The experiments were replicated 3 times, with each aging trial documenting the lifespan of 120 *C. elegans*. **** *p* < 0.0001 versus control. (**B**,**C**) MC-LR exposure had a negative effect on brood size and hatching rate of nematodes. The values are presented as the mean ± SEM and were analyzed using a *t*-test (n = 6 per group). The experiments were replicated 6 times, with each instance documenting the reproductive capacity of 10 *C. elegans*. D1–D7 indicates Day 1–7 (ages). * *p* < 0.05 versus control.

As depicted in Figure 1A, MC-LR markedly reduced the lifespan of nematodes. Furthermore, MC-LR significantly decreased both the egg-laying capacity and hatching rate of nematodes, with consistent observations in daily egg-laying and hatching rate data (Figure 1B,C).

### 2.2. MC-LR Affects the Chemotaxis of Nematodes towards Benzaldehyde

**Figure 2:**
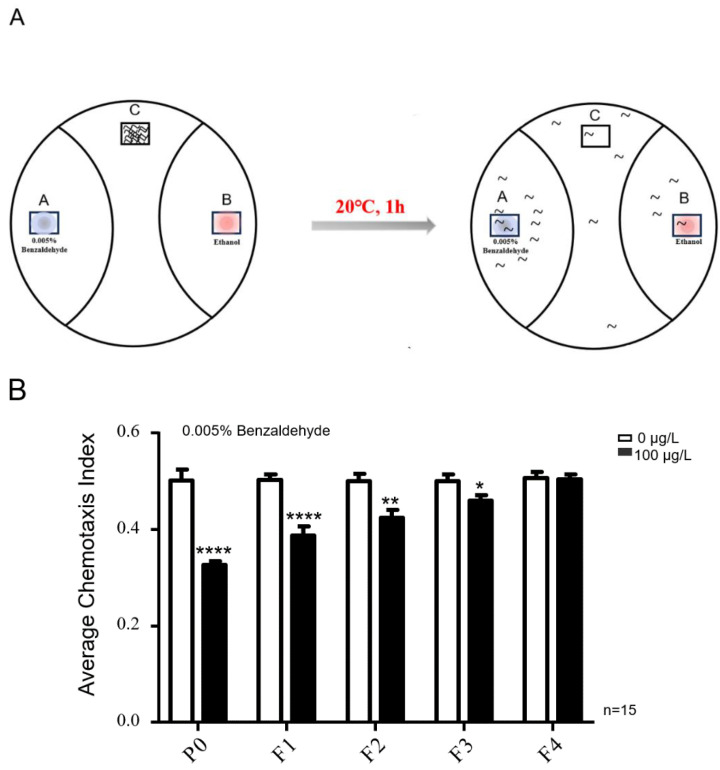
MC-LR affects the chemotaxis of nematodes towards benzaldehyde, transmitting this influence to the F3 generation. (**A**) Schematic representation of the chemotaxis in nematodes. chemotaxis index = (A − B)/(A + B + C). (**B**) The chemotaxis index of nematodes towards 0.005% benzaldehyde following exposure to MC-LR. The values are presented as the mean ± SEM and were analyzed using a *t*-test (n = 15 per group). The experiments were replicated 15 times, with approximately 100 *C. elegans* participating in each trial. **** *p* < 0.0001 versus control. ** *p* < 0.01 versus control. * *p* < 0.05 versus control.

The chemotaxis assays of *C. elegans* are shown in Figure 2A. Exposure to MC-LR significantly reduced the chemotaxis of nematodes towards benzaldehyde, and this effect was transmitted to the F3 generation of nematodes (Figure 2B).

### 2.3. MC-LR Accumulates within the Nematodes

**Figure 3:**
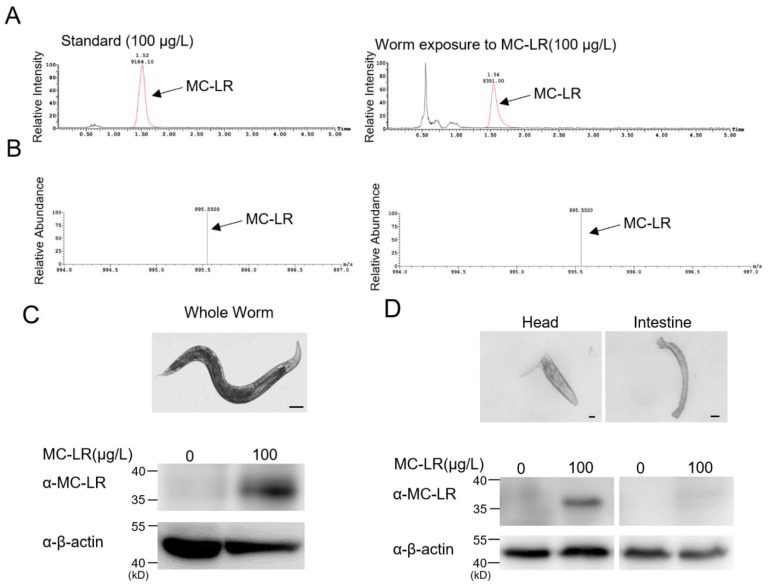
MC-LR primarily accumulates in the head of nematodes. (**A**,**B**) Exposure worm whole-cell lysate was analyzed using mass spectrometry. There was a large accumulation of MC-LR in the exposed nematodes. (**C**,**D**) Immunoblotting analysis used an anti-MC-LR antibody for MC-LR accumulation in the study of different tissues. Scale bars: 100 μm (Whole worm); 10 μm (Head and intestine).

The LC-MS results indicated that MC-LR, after immersion exposure, could accumulate inside the nematodes (Figure 3A,B). To delve deeper into the mechanisms underlying MC-LR neurotoxicity, we conducted dissections on nematodes that had been exposed to MC-LR. Through a Western blot identification of MC-LR in both the intestine and head region, which is rich in neurons, we observed a predominant accumulation of MC-LR in the nematode’s head (Figure 3C,D). This finding suggested that the neurotoxicity of MC-LR may have been a crucial factor, providing valuable insights into the understanding of MC-LR’s neurotoxic effects on other animals.

### 2.4. MC-LR Is Capable of Binding to PP2A-C within Nematodes

**Figure 4:**
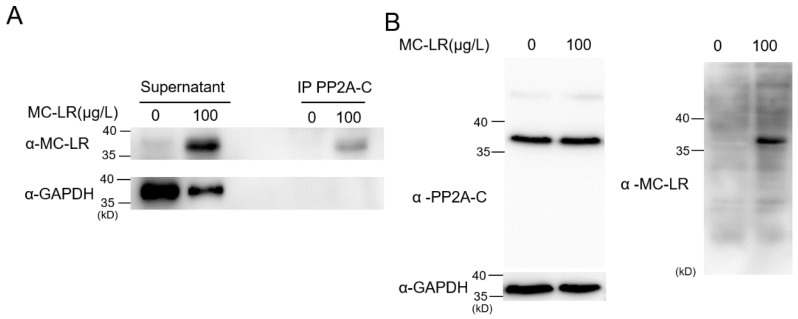
MC-LR is capable of binding to PP2A-C within nematodes. (**A**) Exposure and non-exposure worm whole-cell lysates were immunoprecipitated (IP) using the PP2A C subunit antibody, which also works with the homolog LET-92, and subsequently Western blotted (WB) using anti-MC-LR (top) or anti-GAPDH (bottom) antibodies. Co-IP results shows a strong MC-LR signal in the blot on the exposure sample. (**B**) Applying two different antibodies, anti-C subunit and anti-MC-LR, respectively, in the same blot, showed that only the lysate of the exposure worm had a signal of the same molecular size in both antibodies.

To further ascertain the binding interaction between MC-LR and PP2A-C, immunoprecipitation experiments were conducted. In the immunoprecipitated assay, we used the antibody of MC-LR to pull down LET-92 protein in an exposure worm lysate (Figure 4A). We detected an immunoblotting signal of the same molecular size when using two different antibodies, anti-LET-92 and anti-MC-LR, in the same blot (Figure 4B). Both sets of experimental results indicated that MC-LR could interact with PP2A-C within the nematode body.

### 2.5. Point Mutations in PP2A-C Prevent the Binding of MC-LR to PP2A-C

**Figure 5:**
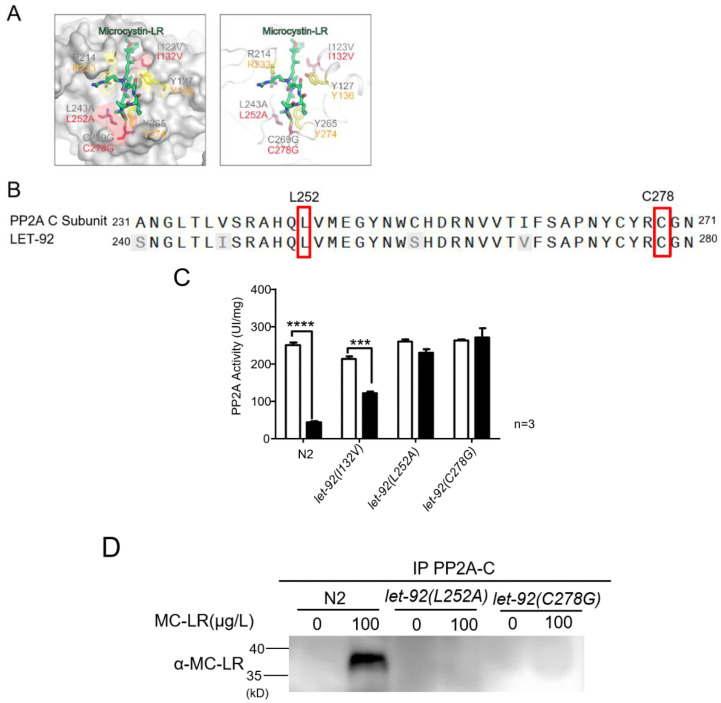
Residues L252 and C278 in LET-92 are required for the binding ability of MC-LR, but do not affect its function. (**A**) Three-dimensional structure diagram of PP2A-C subunit binding with MC-LR. Mammal residues (top); *C. elegans* residues (bottom). L252 and C278 formed a binding-site pocket using van der Waals forces and covalent bonds, respectively, to interact with MC-LR. (**B**) Sequence alignment of mammalian C subunit and *C. elegans* LET-92 homolog. The two L252 and C278 residues, highlighted in the red box, were conserved in both proteins. The shaded regions indicate non-conserved amino acids within the LET-92 protein in *C. elegans.* (**C**) Measurement of PP2A enzyme activity. The values are presented as the mean ± SEM and were analyzed using a *t*-test (n = 3 per group). The experiments were replicated 3 times. *** *p* < 0.001 versus control; **** *p* < 0.0001 versus control. (**D**) L252 and C278 in LET-92 are required for the binding ability of MC-LR. Worm whole-cell lysates were prepared from wild-type and two-point mutation strains. Samples were immunoprecipitated and subsequently Western blotted using PP2A-C subunit and MC-LR antibodies, respectively.

A three-dimensional structure diagram of the PP2A-C subunit showed the binding sites with MC-LR (Figure 5A). Based on the sequence alignment results, it was observed that these critical amino acid residues were highly conserved within the nematodes (Figure 5B). We employed a CRISPR knock-in approach to introduce point mutations to all six sites; three of them had development problems or were lethal at the embryo stage. The rest of the variants (*let-92(I132V)*, *let-92(L252A)*, and *let-92(C278G)*) developed normally and produced adult animals. Following exposure to MC-LR, the PP2A activity in the *let-92(L252A)* and *let-92(C278G)* variants remained unaffected, while the *let-92(I132V)* mutant exhibited a significant reduction in PP2A activity (Figure 5C). L252 and C278 formed a binding-site pocket using van der Waals forces and covalent bonds to interact with MC-LR, respectively. Strikingly, these two functional LET-92 variants were not MC-LR-sensitive after exposure. They had a similar phosphorylation activity compared with the non-exposure worms (Figure 5C). We found that both variant lysates had no anti-MC-LR signal in the immunoblotting assay (Figure 5D).

### 2.6. The Effect of MC-LR on the Response of Point-Mutant Nematodes to Benzaldehyde

**Figure 6:**
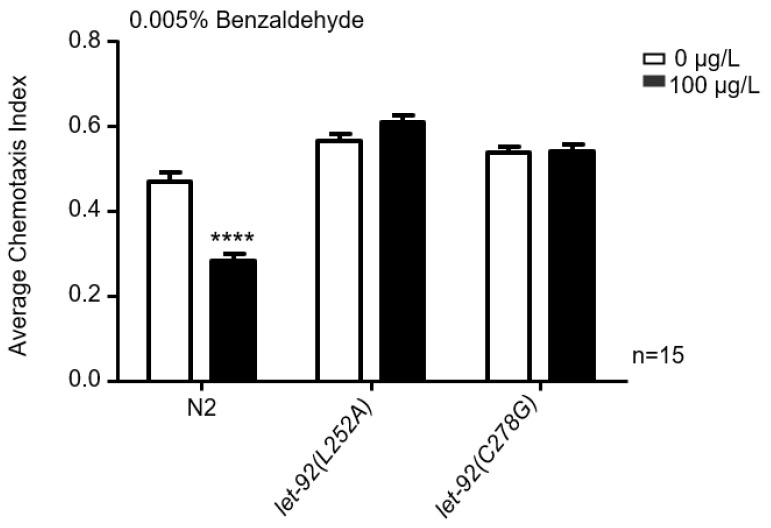
The impact of MC-LR exposure on chemotaxis mediated by *let-92(L252A)* and *let-92(C278G)*. The chemotaxis index of nematodes towards 0.005% benzaldehyde following exposure to MC-LR. The values are presented as the mean ± SEM and were analyzed using a *t*-test (n = 15 per group). The experiments were replicated 15 times, with approximately 100 *C. elegans* participating in each trial. **** *p* < 0.0001 versus control.

As depicted in Figure 6, nematodes exposed to MC-LR exhibited a reduced chemotaxis response to benzaldehyde, whereas *let-92(L252A)* and *let-92(C278G)* exposed to MC-LR did not show any alteration in their chemotaxis response to 0.005% benzaldehyde.

### 2.7. The Impact of MC-LR on Calcium Signaling by Benzaldehyde in Point-Mutant Nematodes

**Figure 7:**
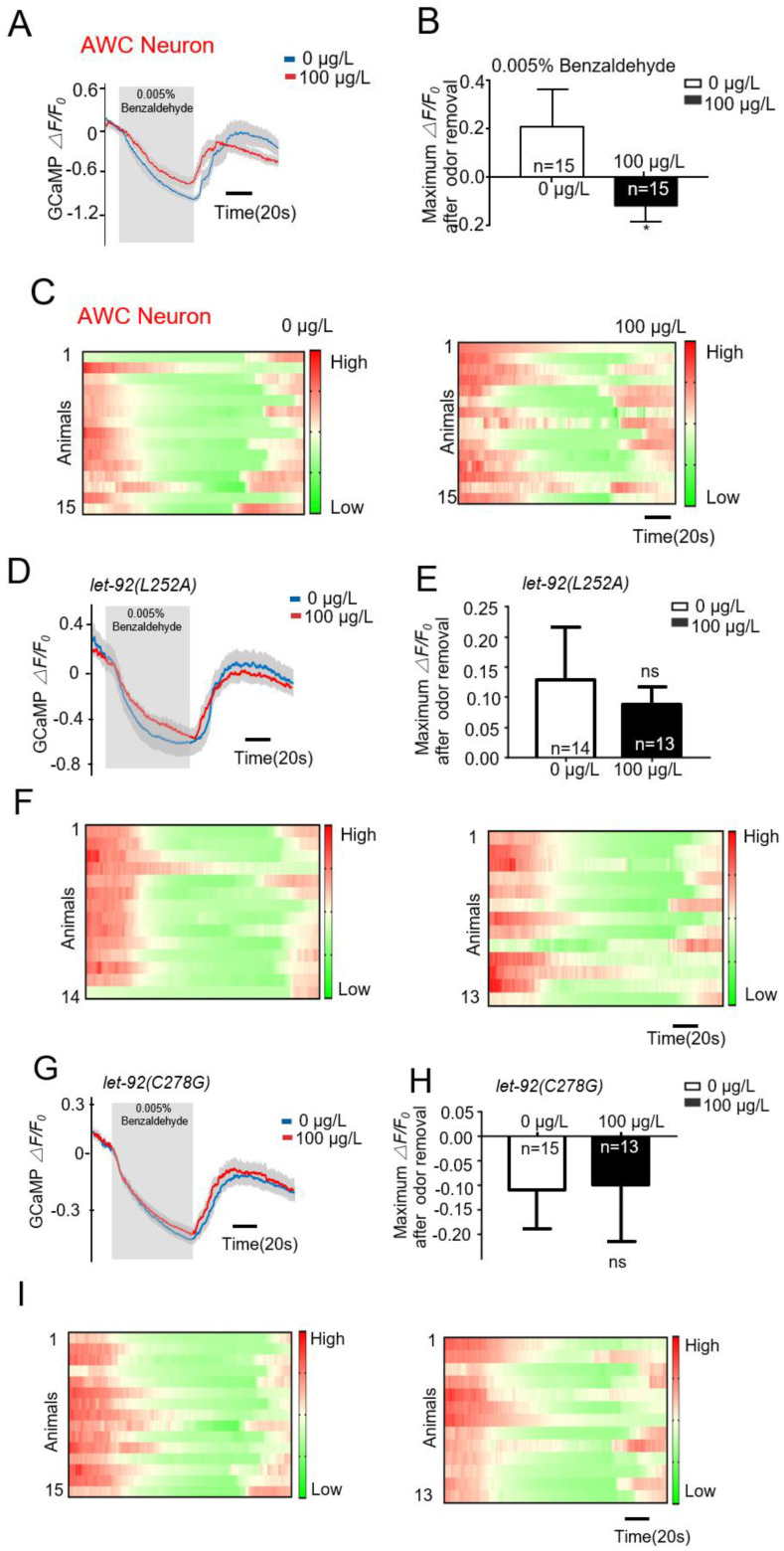
Residues L252 and C278 in LET-92 are required for MC-LR to affect AWC neuronal functions. (**A**–**C**) Repellent and attractive odors evoked a robust calcium response in AWC neurons, which was severely defective in MC-LR exposure worms. (**A**) Average traces with error bars. (**B**) Bar graph. (**C**) Every row in the heatmaps corresponds with images cropped from an individual worm. The color keys in the heatmaps align with the respective response curves. The values are presented as the mean ± SEM and were analyzed using a *t*-test (n = 15 per group; n represents the number of replications in the experiment). * *p* < 0.05 versus control. (**D**−**I**) Repellent odor evoked a robust calcium response in AWC neurons, which were not defective in the two-point mutation strains when exposed to MC-LR. (**D**,**G**) Average traces with error bars. (**E**,**H**) Bar graph. (**F**,**I**) Every row in the heatmaps corresponds with images cropped from an individual worm. The color keys in the heatmaps align with the respective response curves. The values are presented as the mean ± SEM and were analyzed using a *t*-test (n ≥ 13 per group).

Live calcium imaging technology directly reflects the functional integrity of neurons. In this experiment, we employed transgenic techniques to express the calcium indicator protein Gcamp6s in the AWC neurons of nematodes. By monitoring changes in calcium signals in the AWC neurons of nematodes, we assessed the sensitivity of the AWC neurons to benzaldehyde stimulation, thereby elucidating the functional integrity of the AWC neurons. As illustrated in Figure 7A–C, nematodes exposed to MC-LR exhibited a reduced calcium response intensity in the AWC neurons when exposed to benzaldehyde, while *let-92(L252A)* and *let-92(C278G)* exposed to MC-LR did not demonstrate any alteration in their calcium response intensity to benzaldehyde (Figure 7D–I). These results indicated that MC-LR impaired the odor perception function of the AWC neurons in the nematodes, but mutations at the L252 and C278 sites of LET-92 could protect the AWC neurons from damage caused by MC-LR.

## 3. Discussion

MC-LR released from cyanobacteria into water bodies has garnered global attention due to its potent toxicity and widespread distribution [39]. Furthermore, the ability of MC-LR to enter the human body through various means such as drinking water, food, and aerosols has heightened concerns [40,41]. Due to its high toxicity and wide distribution, the WHO has established a maximum allowable concentration of 1 μg/L for MC-LR in drinking water. However, in natural aquatic ecosystems, the concentration of MC-LR can often reach several tens or even hundreds of micrograms per liter [9]. Previous studies have already reported the toxicity of nematodes at lower MC-LR concentrations [42,43,44]. We exposed *C. elegans* to MC-LR at concentrations of 10, 50, 100, 500, and 1000 μg/L and evaluated the effect of MC-LR on the avoidance of aversive odors of *C. elegans* (data not shown). The results indicated that concentrations of 10 and 50 μg/L MC-LR had no discernible effect on nematode avoidance of aversive odor. It is noteworthy that our experimental results may have been influenced by the fact that the exposure of the nematodes to MC-LR was brief. In contrast, in the natural environment, wildlife is subject to prolonged exposure to varying concentrations of MC-LR, posing higher risks [45]. In consideration of our data and to facilitate the further exploration of the neurotoxic mechanisms of MC-LR, we exposed nematodes to a concentration of 100 μg/L MC-LR in our experiments.

Acute exposure to MC-LR may result in a range of symptoms affecting the digestive, respiratory, and inflammatory systems such as paralysis and respiratory or cardiac arrest, which can ultimately lead to mortality [46,47]. Prolonged exposure is linked to elevated incidences of various cancers, including (but not limited to) liver, brain, kidney, and ovarian cancer as well as leukemia and others [48]. It has been recognized that MC-LR not only exhibits hepatotoxicity and reproductive toxicity but also causes varying degrees of damage to the respiratory, digestive, and nervous systems in animals. Field investigations and laboratory findings have both shown that in addition to substantial accumulations in the livers of animals, MC-LR also significantly accumulates in the gonads [49,50,51]. A recent study indicated that MC-LR accumulation in zebrafish larvae results in reduced cholinergic system activity, primarily characterized by decreased levels of dopamine (DA) and acetylcholinesterase, along with increased acetylcholinesterase activity, leading to reduced muscle contractions and behavioral responses [52]. Our study revealed the accumulation of MC-LR in the head region of nematodes, particularly in areas rich in neurons. This finding suggests that the neurotoxicity of MC-LR may be a crucial factor, providing valuable insights into the understanding of MC-LR’s neurotoxic effects on other animals. In this study, we comprehensively assessed the toxicity effects of MC-LR, utilizing the advantages of nematodes. Our results indicated that MC-LR significantly reduced the nematode lifespan and profoundly affected their reproductive and sensory capabilities. These data highlight the comprehensive toxic effects of MC-LR on organisms.

A recent investigation demonstrated that the prolonged exposure of adult zebrafish to environmentally relevant concentrations of MC-LR (1–25 μg/L) resulted in toxin accumulation and developmental neurotoxicity in subsequent generations [53]. However, due to the longer life cycle of zebrafish, it is challenging to perform multi-generation tracking. Given the advantages of *C. elegans* such as simple genetic manipulation, a short lifespan, and well-established behavioral models, it is now widely applied in toxicology research [54]. Our findings revealed, for the first time, that exposure to MC-LR resulted in a decreased ability of nematodes to perceive odors, and this phenotype persisted up to the F3 generation. The ability of animals to perceive odors is crucial for their survival as well as foraging, reproduction, and other essential biological behaviors [55]. Our data suggest that the neurotoxic effects of MC-LR can persist up to the F3 generation, posing a significant threat to the population.

Individuals suffer from the toxicity of MC-LR by consuming water and food exposed to MC-LR [3]. A recent study indicated that MC-LR can disperse over long distances in the air through aerosols, suggesting that respiratory and dermal exposure are additional pathways for MC-LR to enter the human body [26]. The half-life of MC-LR in its normal state is approximately 7 days. However, when it forms complexes with other substances such as microplastics, the degradation of MC-LR is significantly prolonged [56]. Upon introduction into the human body, MC-LR undergoes systemic distribution through the bloodstream, reaching diverse tissues and organs. Subsequently, it permeates cells through organic anion transporting polypeptides (OATPs), inducing toxic effects at the cellular level. One of the most crucial pathways of MC-LR toxicity is its specific binding to the C subunit of PP2A, resulting in the deactivation of PP2A. PP2A is indispensable for the proper operation of numerous signaling pathways, especially those governing cell processes like cell division, regulation of the cell cycle, response to DNA damage, stress response (e.g., hypoxia), reactions to growth factors, cell adhesion, viability, and various facets of cellular fate encompassing apoptosis, autophagy, neuronal signaling, and brain development [57,58,59]. The homologous protein of PP2A-C in nematodes, LET-92, shares over 90% amino acid sequence conservation [60]. In preceding structural biology investigations, it has been demonstrated that MC-LR binds to the active site pocket of the PP2A catalytic subunit. This pocket, situated above two manganese atoms and the active site of PP2A, forms a robust interaction with MC-LR through a covalent bond between the Sγ atom of Cys269 and the terminal carbon atom of Mdha. Towards one extremity of the binding pocket, several amino acids of the PP2A catalytic subunit-namely, Gln122, Ile123, His191, and Trp200-construct a hydrophobic cage to accommodate the extended hydrophobic Adda side chain of MC-LR. Conversely, at the opposite end of the binding pocket, Leu243, Tyr265, Cys266, Arg268, and Cys269 partake in multiple van der Waals interactions with the hydrophobic segment of MC-LR. Furthermore, Arg89 establishes two hydrogen bonds with distinct oxygen atoms in the binding of MC-LR, while Tyr265 similarly contributes hydrogen bonds to the binding of MC-LR [61]. However, the specific roles of these key amino acid binding sites regarding MC-LR binding to PP2A-C have not been thoroughly investigated. We conducted in-depth research into the effects of single amino acid mutations on PP2A activity and its binding ability with MC-LR using gene-edited nematodes. Our results indicated that the I132 amino acid mutation led to a reduction in PP2A-C activity, while the L252 and C278 amino acid mutations prevented the binding of PP2A-C with MC-LR without affecting PP2A activity. This discovery was validated through nematode behavioral studies and in vivo calcium imaging.

Since the WHO established the drinking water standard for MC-LR in 2006, sporadic cases of human poisoning due to MC-LR continue to occur worldwide [62]. In 2007, an Argentine male experienced MC-LR poisoning after surfing in a water body affected by a bloom of cyanobacteria, ingesting water contaminated with approximately 48.6 μg/L of MC-LR. Three days later, the patient exhibited gastrointestinal distress and pneumonia, followed by hepatotoxicity a week later [23]. Beyond acute poisoning, prolonged exposure to MC-LR poses greater risks to human health. Chen et al. documented a correlation between the detection of MC-LR in the serum samples of fishermen from Chaohu Lake in China and the occurrence of liver damage [24]. An analysis of MC-LR in vegetables and aquatic products in Nanjing, China, revealed a positive correlation between MC-LR exposure and the risk of prostate cancer [63]. Our experimental findings suggested a potential avenue: the development of competitive small molecules targeting two key amino acid sites on PP2A inhibiting the binding of MC-LR to PP2A. This approach could be dual-purpose, providing a therapeutic option for individuals suffering from acute poisoning while also serving as a basis for the development of health supplements to protect those experiencing prolonged exposure to MC-LR.

## 4. Conclusions

In summary, our results demonstrate that MC-LR affected the lifespan, reproduction, and sensory capabilities of nematodes, indicating that its toxic effects on organisms are comprehensive. Additionally, for the first time, we observed a significant reduction in nematode sensory capabilities caused by MC-LR exposure. This neurotoxic effect persisted up to the F3 generation. This suggests a significant threat to biological populations in natural environments due to MC-LR. Furthermore, through gene editing and in vivo calcium imaging, we identified two crucial amino acid sites on PP2A-C. Mutations at these two amino acid sites prevented the binding of MC-LR to PP2A-C without affecting its activity. In conclusion, we discovered that the neurotoxic effects of MC-LR can be transmitted to the F3 generation, and we identified two amino acid sites on PP2A-C that could prevent its binding to MC-LR, laying the foundation for the development of MC-LR detoxification drugs.

## 5. Materials and Methods

### 5.1. C. elegans Strains

*C. elegans* strains (Table 1) were nurtured at a temperature of 20 °C on plates containing a nematode growth medium (NGM) with OP50 bacteria as the seeding source. The creation of transgenic lines involved the direct injection of plasmid DNA into the gonads of hermaphrodites.

### 5.2. Experimental Design

MC-LR with a purity level of 95% (1 mg) was procured from the Taiwan Algae Science Company. A solution containing MC-LR was prepared by adding 1 mL ultrapure water, resulting in a concentration of 1 mg/mL. Gravid hermaphrodites were lysed to release eggs and then transferred to 3 mL of an S medium containing (0, 100 μg/L) MC-LR for liquid culture using a 6-well plate with support from concentrated OP50 bacteria. *C. elegans* were cultured in 6-well plates until the first day of adulthood, then they were collected for this experiment. All additional reagents employed in this study were of analytical grade. We harvested eggs from parental nematodes subjected to MC-LR exposure and nurtured them in liquid media devoid of MC-LR until adulthood, at which point they were utilized for the behavioral assays. Simultaneously, we collected eggs again and continued culturing them under MC-LR-free conditions until the F4 generation of the nematodes.

### 5.3. Behavioral Assays

The chemotaxis assays of *C. elegans* are shown in Figure 2A. Gravid hermaphrodites were lysed to release eggs and then transferred to 3 mL of an S medium containing (0, 100 μg/L) MC-LR for liquid culture using a 6-well plate with support from concentrated OP50 bacteria. *C. elegans* were cultured in 6-well plates until the first day of adulthood, then they were collected for this experiment. Following exposure to MC-LR, the worms underwent two washes with an M9 buffer. Subsequently, chemotaxis assays were conducted using assay agar in 90 mm Petri dishes. The odorant benzaldehyde (0.005%) was diluted in ethanol as the worms exhibited no attraction to ethanol. At the control and odor point sources, the worms were immobilized with 1 microliter of 2% sodium azide, followed by the application of 1 microliter of the diluted odor or a control (ethanol). Adult worms were strategically placed at the origin and positioned equidistantly from both the control and odor points along the plate’s edge. After 1 h at 20 °C and 4 h at 4 °C, the enumeration of worms at the odor, control, and midpoint locations was conducted. We collected eggs from parent nematodes exposed to MC-LR and cultured them in liquid media without MC-LR until they reached adulthood for the behavioral assays. Simultaneously, we collected eggs again and continued culturing them under MC-LR-free conditions until the F4 generation of nematodes.

### 5.4. CRISPR/Cas9-Mediated Genome Editing

*let-92(L252A)*, *let-92(I132V)*, and *let-92(C278G)* were generated by SunyBiotech upon our request. sgRNA was designed according to the required mutant genes, and Cas9 sgRNA expression plasmids were constructed. The constructed plasmids were injected into the gonad using a microinjection. PCR and sequencing were used to determine if the gene was successfully recombined and if it had mutated.

### 5.5. Lifespan and Brood Assay

All longevity assessments were carried out at a temperature of 20 °C. The initiation of adulthood was consistently designated as day 1 across all experiments. Each lifespan trial encompassed 120 worms, with regular transfers every 2–3 days to fresh 60 mm NGM plates at a density of 12 worms per plate. Worms were censored if they wandered off the plate or experienced explosive or bagging events. MC-LR (100 μg/L) was supplemented in NGM plates. Statistical analyses were executed using GraphPad Prism 5 (GraphPad Software, Inc. California, USA), with *p*-values determined using the log-rank (Kaplan–Meier) method. All lifespan experiments underwent replication on at least two separate occasions. After exposure to MC-LR, the nematodes were subjected to triple washes with an M9 buffer and were subsequently distributed onto NGM plates with a diameter of 30 mm and one nematode per plate. A minimum of 10 plates were utilized for each brood assay experiment. Egg production and hatching rates were quantified daily, and the entire experiment was replicated six times.

### 5.6. Biochemistry

Total protein extracts were prepared using a lysis buffer. Protein extracts underwent separation using SDS-PAGE gels and their subsequent transfer onto a polyvinylidene difluoride membrane (Millipore). Immunoblotting was then performed using the specified primary antibodies. Antibodies against MC-LR (1:1000; ALX-804-320) were obtained from Enzo Life Science. Antibodies against PP2A-C subunit (1:1000; #2038) were obtained from Cell Signaling. Antibodies against GAPDH (1:1000; GB11002) were obtained from Servicebio.

The anti-PP2A-C subunit antibody (1:1000; #2038) was dialyzed against a coupling buffer and immobilized on 1 mL carboxyl-activated MagPoly beads (Changzhou Smart-Lifesciences Biotechnology, Changzhou, China). The antibody concentration was 2 mg/mL of the coupling buffer. The nematode lysate was incubated with the magnetic beads overnight at 4 °C. The protein bound to the magnetic beads was eluted with 0.1 M of a glycine solution. Concentrated protein samples underwent the processes of SDS-PAGE and Western blotting.

### 5.7. LC-MS/MS Analysis

The harvested nematodes (n = 3) underwent measurements and weighing. Subsequently, the samples were lyophilized using liquid nitrogen. After extraction, purification, and drying, the samples were reconstituted in 100 µL of the mobile phase.

An LC-MS/MS analysis was conducted utilizing a Xevo TQ-XS mass spectrometer linked to an Acquity UPLC-Class System (Waters). Confirmation of peak time was achieved using the multiple reaction monitoring (MRM) mode with precursor and fragment ions at *m*/*z* 498.61–135.11 and *m*/*z* 498.61–861.27, respectively. The capillary voltage was set at 3.5 kV and the desolation temperature was maintained at 350 °C. Positive ion mode mass scan spectra were acquired over a range of *m*/*z* 450–1000. The capillary voltage was adjusted to 1.5 kV and the cone voltage to 8V. An MS2 scan analysis of the *m*/*z* 498.3 molecule was performed in a molecular weight range of *m*/*z* 100–400 with a capillary voltage of 1.5 kV, a cone voltage of 15 V, and a collision energy of 35 eV. Stock solutions (1 × 10^6^ μg/L) were prepared by dissolving MC-LR in pure water. Standard solutions (0, 1, 10, 50, and 100 μg/L) were prepared using serial dilutions of stock solutions with pure water. We used the mass spectrometry detection condition to detect the MC-LR standard solution and prepare a standard curve. The regression coefficient was R^2^ = 0.9393, indicating that this condition could be used for the detection of MC-LR in the nematode samples.

### 5.8. PP2A Activity Analysis

PP2A enzymatic activity was assessed using the Serine/Threonine Phosphatase Assay System (Shanghai Enzyme-linked Biotechnology, YJ023419; China), following the provided instructions. For the assay, the wells were initially loaded with samples or standards; subsequently, the antibody mix was introduced. Following incubation, thorough washing was carried out to eliminate any unbound material. The addition of the TMB Development Solution initiated a catalytic reaction by HRP during incubation, resulting in the development of a blue color. The color change from blue to yellow was completed by halting the reaction with the addition of a Stop Solution. The measured intensity at 450 nm was directly proportional to the quantity of the bound analyte, generating the corresponding signal.

### 5.9. Calcium Imaging

Ca^2+^ activities of AWC neurons were visualized using strains expressing GCaMP6.0 driven by the *odr-1* promoter. The animals were immersed in a bath solution. Subsequently, they were affixed to a glass coverslip using a medical-grade cyanoacrylate-based glue (Gluture Topical Tissue Adhesive, Abbott Laboratories). A 0.005% vol/vol dilution of benzaldehyde served as the stimulus. For the calcium imaging of AWC neurons, we initially recorded 50 s of baseline activity in the bath solution, followed by 75 s of exposure to the benzaldehyde stimulus, and finally, 2 min of a buffer-only observation. An Olympus microscope (IX71) with a 40× objective lens and PRIME camera was employed to acquire fluorescent images. The data collection utilized VisiView software 1, and the peak percentage change in GCaMP fluorescence intensity (ΔF/F_0_) was quantified.

### 5.10. Statistical Analysis

We employed AI (ChatGPT 3.5) to make appropriate modifications to the manuscript. The statistical results were analyzed and processed using GraphPad Prism 8. The statistical data were presented as the mean ± SEM and the comparison between the two groups of data adopted the unpaired two-tailed *t*-test. *p* < 0.05 was considered to be statistically significant. When the *p*-value was less than 0.05, there was a significant difference, which was recorded as *. *p* < 0.01 was marked as **, *p* < 0.001 was marked as ***, and *p* < 0.0001 was marked as ****.

## Figures and Tables

**Table 1:** All *C. elegans* strains used in the experiment.

Strains	Source	Identifier
N2	CGC (University of Minnesota-Twin Cities, Minneapolis, MN, USA)	
*let-92(syb6505[C278G])*	SunyBiotech (FuZhou, China)	PHX6506
*let-92(syb6455[L252A])*	SunyBiotech	PHX6455
*let-92(syb6542[I132V])*	SunyBiotech	PHX6542
*Ex [Podr-1:Gcamp6s+Pstr-2:mNepturn]*	Yanxun V. Yu	YVY150
*gonEx 111 [Podr-1:Gcamp6s+Pstr-2:mNepturn];let-92(syb6505)*	This study	JKG 712
*gonEx 112 [Podr-1:Gcamp6s+Pstr-2:mNepturn];let-92(syb6455)*	This study	JKG 713

## Data Availability

The data presented in this study are available on request from the first author.
